# Chlorophyll Deficiency in the Maize *elongated mesocotyl2* Mutant Is Caused by a Defective Heme Oxygenase and Delaying Grana Stacking

**DOI:** 10.1371/journal.pone.0080107

**Published:** 2013-11-11

**Authors:** Dianyi Shi, Xu Zheng, Liang Li, Wanhuang Lin, Wenjun Xie, Jianping Yang, Shaojiang Chen, Weiwei Jin

**Affiliations:** 1 National Maize Improvement Center of China, Key Laboratory of Crop Genetic Improvement and Genome of Ministry of Agriculture, China Agricultural University, Beijing, China; 2 National Key Laboratory of Plant Molecular Genetics, Institute of Plant Physiology and Ecology, Shanghai Institutes for Biological Sciences, Chinese Academy of Sciences, Shanghai, China; 3 Institute of Crop Sciences, Chinese Academy of Agricultural Sciences, Beijing, China; 4 Hunan Provincial Key Laboratory of Phytohormones and Growth Development, Hunan Agricultural University, Changsha, China; Key Laboratory of Horticultural Plant Biology (MOE), China

## Abstract

**Background:**

Etiolated seedlings initiate grana stacking and chlorophyll biosynthesis in parallel with the first exposure to light, during which phytochromes play an important role. Functional phytochromes are biosynthesized separately for two components. One phytochrome is biosynthesized for apoprotein and the other is biosynthesized for the chromophore that includes heme oxygenase (HO).

**Methodology/Principal Finding:**

We isolated a *ho1* homolog by map-based cloning of a maize *elongated* *mesocotyl2* (*elm2*) mutant. cDNA sequencing of the *ho1* homolog in *elm2* revealed a 31 bp deletion. De-etiolation responses to red and far-red light were disrupted in *elm2* seedlings, with a pronounced elongation of the mesocotyl. The endogenous HO activity in the *elm2* mutant decreased remarkably. Transgenic complementation further confirmed the dysfunction in the maize *ho1* gene. Moreover, non-appressed thylakoids were specifically stacked at the seedling stage in the *elm2* mutant.

**Conclusion:**

The 31 bp deletion in the *ho1* gene resulted in a decrease in endogenous HO activity and disrupted the de-etiolation responses to red and far-red light. The specific stacking of non-appressed thylakoids suggested that the chlorophyll biosynthesis regulated by *HO1* is achieved by coordinating the heme level with the regulation of grana stacking.

## Introduction

Chlorophyll (Chl) plays a central role in the harvesting of light energy for photosynthesis. The regulation of Chl metabolism includes all levels of control to balance the metabolic flow in response to external and endogenous variation during plant development [1-5]. In the dark, germinating seedlings utilize all of the nutrients contained in the seeds to establish conditions suited for harvesting light signals. This results in the dramatic elongation of the hypocotyl. Within the cotyledons, proplastids differentiate into etioplasts. A large supply of the Chl precursor protochlorophyllide (Pchlide) is also built up in the prolamellar bodies [1,6-9].

During the first exposure to light, etiolated seedlings initiate grana stacking and reduce the accumulated Pchlide to Chl; these changes occur in parallel and collectively lead to rapid greening [6,10]. This process is called photomorphogenesis [11,12], during which phytochromes play an important role and are solely responsible for the perception of red and far-red light [13-15]. In Arabidopsis, the phytochrome family consists of five genes, *PHYA-E* [16]. Most monocots typically have the following three phytochromes: *PhyA*, *PhyB*, and *PhyC* [15,17]. In maize, an ancestral genomic duplication has enlarged the total family size to at least six genes [18-22].

Holophytochrome biosynthesis requires the convergence of two separate pathways, one for synthesis of the apoprotein and another for the synthesis of the chromophore phytochromobilin (PΦB) [21]. Heme oxygenase (HO; EC1.14.99.3) belongs to the PΦB synthetic branch and is responsible for oxidizing heme to biliverdin (BV) [12,23-28]. BV is then reduced to PΦB by PΦB synthase and is subsequently isomerized [29]. Although phytochrome apoproteins are encoded by a multigene family, it is likely that all plant apophytochromes bind the same chromophore [30,31]. Therefore, the genetic disruption of chromophore PΦB synthesis could inactivate the entire phytochrome system. Such disruption usually leads to a yellowish phenotype. There are a number of known mutants in which the synthesis of chromophore PΦB is disrupted, such as the Arabidopsis mutants *hy1* and *hy2* [25,26,32,33], the tobacco (*Nicotiana plumbaginifolia*) mutants *pew1* and *pew2* [34], the pea (*Pisum sativum*) mutants *pcd1* and *pcd2* [24,35], the tomato (*Lycopersicon esculentum*) mutants *au* and *yg-2* [23,36], the rice mutant *se-5* [27,37], and the maize mutant *elm1* [38,39]. All of the above mutants are defective in either HO or PΦB synthesis.

There are four putative *HO* genes in Arabidopsis; these genes are known as *HY1* (*AtHO1*), *AtHO2*, *AtHO3* and *AtHO4*. Genetic analysis demonstrated that *HY1* is responsible for the majority of BV synthesis; *hy1* mutant plants have long hypocotyls and decreased accumulation of chlorophyll [25,26]. Further research indicated that family members other than the *AtHO1* gene also play a role in synthesizing BV during photomorphogensis, but the effects of these genes are subtler [12,28]. Inactivation of HO1 in the tomato *yg-2* mutant enhances the heme level; this post-translationally inhibits the first enzyme committed to tetrapyrrole biosynthesis, Glu-tRNA reductase (designated HEMA) and decreases Chl biosynthesis [40,41]. HO is a mutifunctional enzyme that is involved in many biological processes. Beside its role in oxidizing heme, this protein also participates in programmed cell death (PCD) [42], adventitious root formation [43-45], and protection from oxidative damage [46-51].

In our current research, we isolated a *ho1* homolog by map-based cloning in the maize *elongated mesocotyl2* (*elm2*) mutant. *elm2* seedlings displayed a disruption in de-etiolation responses under red and far-red light conditions, with a pronounced elongation of the mesocotyl. The endogenous HO activity in *elm2* decreased remarkably. The chloroplast ultrastructure showed that the *elm2* mutant was delayed in the stacking of the grana but not in the stacking of the non-appressed thylakoids in the seedling stage. In conclusion, we believe that *ELM2* encodes a *HO1* homolog in maize. The specific stacking of non-appressed thylakoids in the *elm2* mutant suggests that chlorophyll biosynthesis is regulated by *HO1* via the coordination of the heme level with the regulation of grana stacking.

## Materials and Methods

### Plant materials and the mapping population

The maize *elm2* mutant is a spontaneous yellow-green leaf mutant isolated from an open-pollinated population. This mutant was self-pollinated for more than 8 generations before mapping based cloning was undertaken. The F_2_ mapping population was generated by crossing the *elm2* mutant with the normal green Zheng58 maize inbred line. In total, 8,350 recessive individuals from the F_2_ generation with a yellow-green phenotype were selected for mapping.

### Marker development, gene annotation and cDNA sequencing

The well-developed SSR markers were obtained from the Maize Genetics and Genomics Database (http://www.maizegdb.org/). To narrow down the region of the targeted locus, BAC sequences on chromosome 9 were used for developing new SSR, InDel and CAPS markers (Table S1). SSR sequences were analyzed using SSRHunter 1.3 software. InDel and CAPS markers were developed by comparing *elm2* and Zheng58 sequences after PCR amplification and sequencing.

Gene annotation within the located region was obtained from the Maizesequence Database (http://www.maizesequence.org). cDNA sequences of the *HO1* homolog from the wild-type line Zheng58 and the mutant *elm2* were amplified with the forward primer 5’-GTCGCTTCCCCGGCACCGTAC-3’ and the reverse primer 5’-CTCACCAGTAATAAAGTTTTAACAG-3’. The resulting amplicons were sequenced and compared. All of the above primers were designed using Primer 5.0 software.

### Transmission electron microscopy analysis

Leaf samples from the wild-type Zheng58 inbred line and the *elm2* mutant were harvested from 4-week-old seedlings and 2-month-old adults grown under natural conditions. Only the top fully expanded leaves (except the flag leaf) were taken as samples. Fresh leaves were quickly sliced into strips with a width of 2 mm. The strips were then fixed in a 3% glutaraldehyde solution containing 0.1 M potassium phosphate (pH 7.2) and further fixed in 1% OsO_4_. The samples were then dehydrated in an ethanol series and embedded in Spurr resin prior to thin sectioning [52,53]. After staining with lead citrate, the samples were examined using a transmission electron microscope (JEM-1230; JEOL).

### Mesocotyl measurement and RT-PCR analysis

Seeds were grown in nutrient soil in artificial-climate chambers under continuous light or in constant darkness at 28°C for 7 d. The light intensities were set to 100 μmol m^-2^ s^-1^ for white light, 30 μmol m^-2^ s^-1^ for red light, 2.5 μmol m^-2^ s^-1^ for far-red light, and 10 μmol m^-2^ s^-1^ for blue light [38]. On day 7, the parts above the mesocotyl were sampled for RNA isolation and cDNA synthesis before RT-PCR analysis [52,53]. Meanwhile, the mesocotyl length was measured to nearest millimeter.

Total RNA was isolated from 100 mg (fresh weight) of maize seedling tissue from above the mesocotyl using an RNA Isolation Kit (Tiangen Biotech). High-quality first-strand cDNA was generated from 5 μg total RNA using Invitrogen's cDNA Synthesis Kit. Specific primers were designed using Primer 5.0 and were used for RT-PCR analysis (Table S2). PCR was performed using 2 µl of a 5-fold dilution of the cDNA, 1 µl each of 10 pmol forward and reverse primer solutions, and 1 unit of Taq polymerase (TransGen Biotech) in a 20 µl reaction volume. The relative abundance of *β-actin* was used as an internal standard.

### Chloroplast isolation and heme oxygenase assay

The seeds were grown in nutrient soil in artificial-climate chambers under continuous 100 μmol m^-2^ s^-1^ white light at 28°C for 10 d. Approximately 0.3 g of the top well-expanded leaves were used for chloroplast isolation according to the methods of Muramoto et al. [26], Balestrasse et al. [54], and Xuan et al. [43] with minor modifications. 

An HO assay was performed as previously described with minor modifications [26,43,55]. The reaction was initiated by adding NADPH. One unit of activity was calculated as the quantity of enzyme needed to produce 1 nM BV per 30 min at 37°C.

### Transgenic complementation

For transgenic identification, the *Elm2* and *elm2* genes were transformed into the Arabidopsis *ho1* mutant *hy1-100* using a pCAMBIA3301 plasmid; this was performed because *Agrobacterium*-mediated transformation is difficult in maize. This plasmid contains kanamycin and phosphinothricin resistance for bacteria selection and plant selection, respectively. The transformed gene is driven by a CaMV 35S promoter. The full-length cDNA fragments encoding *Elm2* and *elm2* were obtained by PCR amplification using the forward primer 5’-AGATATCGTCCGTCATCGGTGCCGTCG-3’ and the reverse primer 5’-CGGATCCCCCCCTCACCAGTAATAAAG-3’. The genes and the vector were digested with EcoRV and BamHI before sub-cloning. The Agrobacterium strain GV3101 harbored the constructs and was used to transform the Arabidopsis *hy1-100* mutant. Homozygous transgenic lines of the T3 generation were used for the comparison of the hypocotyl length. 

### Determination of pigments

Sampling for the Chl and carotenoid (Car) determination was performed in parallel with the transmission electron microscopy analysis. Total Chl and Cars were extracted with 80% acetone and detected with DU 800 UV/Vis Spectrophotometers (Beckman Coulter). Total Chl and total Cars were calculated according to the absorbance of the extract at 663 nm, 646 nm and 470 nm using equations established by Lichtenthaler and Wellburn [56]: Ca=12.21A663-2.81A646, Cb=20.13A646-5.03A663, and Cx+=(1000A470-3.27Ca-104Cb)/229). 

### Accession Numbers

Sequence data from this article can be found in the GenBank/EMBL data libraries under the following accession numbers: AtHO1 (AT2G26670), AtHO2 (AT2G26550), AtHO3 (AT1G69720), AtHO4 (AT1G58300), SbHO1 (XP_002438642), OsHO1 (NP_001058011), B73-Chr9 (KC404968), B73-Chr6 (AFW76417), Zheng58-Chr9 (KC404967), Zheng58-Chr6 (KC731572), *elm2*-Chr9 (KC404965), *elm2*-Chr6 (KC404966), and EU962994.

## Results

### Genetic characterization of the *elm2* mutant

**Figure 1:**
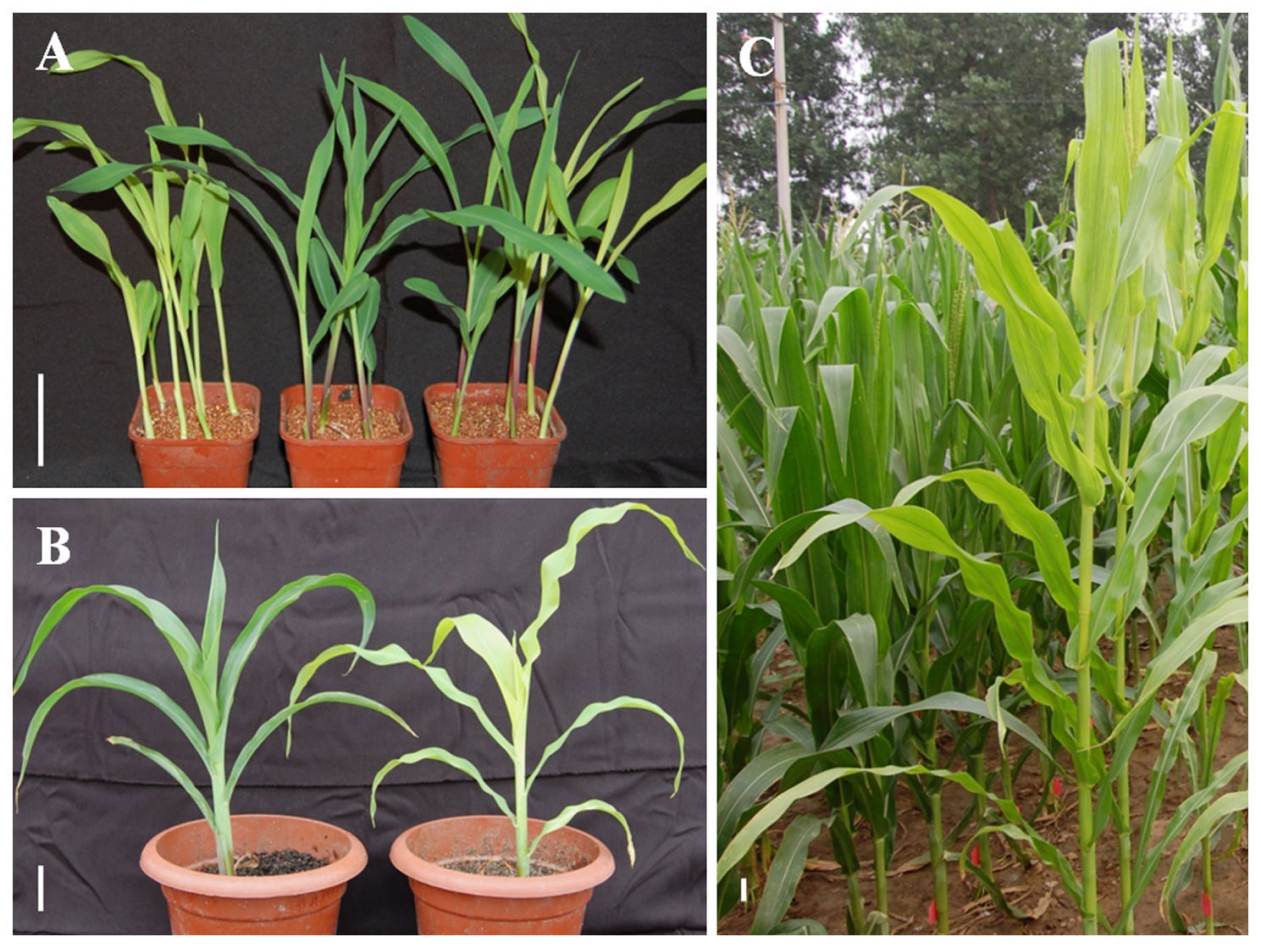
Phenotypes of the wild-type line Zheng58, the mutant *elm2*, and the F_2_ plants. A, 10 d *elm2* mutant seedlings (left), Zheng58 wild-type seedlings (middle), and F_2_ seedlings (right). B, 4-week-old Zheng58 seedlings (left) and *elm2* seedlings (right). C, 2-month-old Zheng58 adult plants (left) and *elm2* adult plants (right) at the tasseling stage. Scale bar=5 cm.

**Figure 2:**
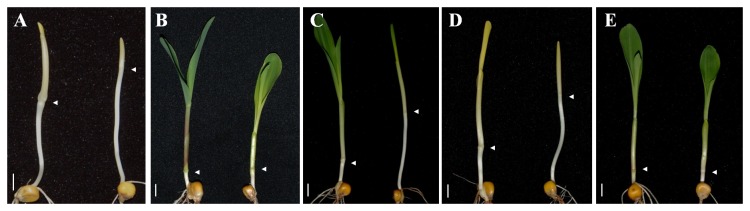
De-etiolation responses in Zheng58 wild-type plants and *elm2* mutants. Representative seedlings were photographed after 7 d of growth in continuous darkness (A), white light (B), red light (C), far-red light (D) or blue light (E) conditions. Left, Zheng58 wild-type seedlings; Right, *elm2* mutants. Scale bar=1 cm.

**Figure 3:**
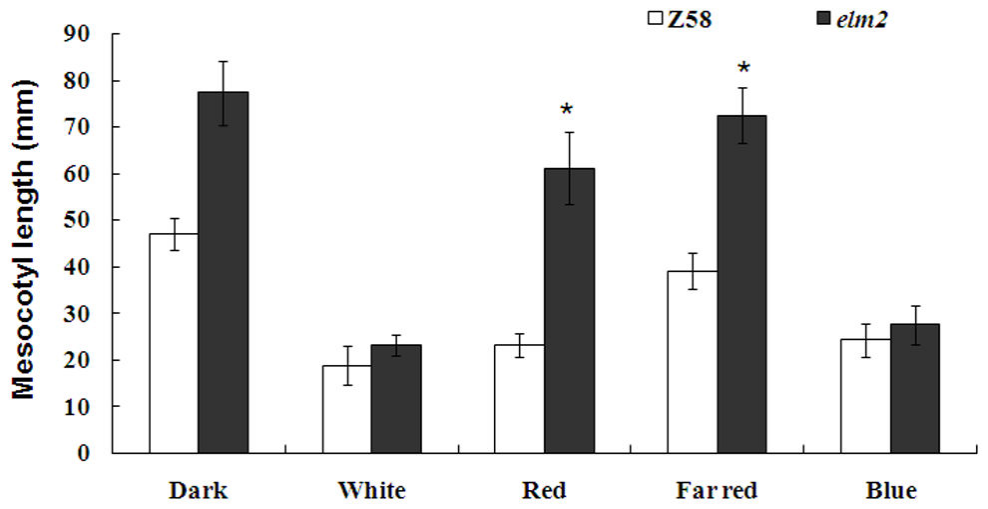
Statistical measurement of mesocotyl length in Zheng58 wild-type plants and *elm2* mutant plants. Zheng58 wild-type seedlings and mutant *elm2* seedlings were grown for 7 d in continuous darkness, white light, red light, far-red light, or blue light conditions. The sample size was 16-18 seedlings per treatment/genotype. Asterisk indicates significant difference as compared with Zheng58 wild-type at *P*<0.01 (Student’s *t* test).

The *elm2* mutant was a spontaneous mutant with yellow-green leaves; this mutant was isolated from an open-pollinated population (Figure 1). The mutant line was self-pollinated for more than 8 generations before the following research was undertaken. In addition to yellow-green leaves, the mutant also presented with an elongated mesocotyl under continuous red or far-red light conditions (Figure 2C, D, 3). A similar phenotype in maize was first reported by Sawers et al. [38]; accordingly, we designated the current mutant as *elm2*. 

All crosses of *elm2* to the wild-type inbred lines Zheng58, Mo17 and B73 yielded only wild-type F_1_ plants. The F_2_ segregation ratio of crosses with Zheng58 and Mo17 tended to be 3:1 (green: yellow-green plants), while the ratio of crosses with B73 tended to be 15:1 (as tested by Chi-square, χ^2^<χ^2^_(0.05, 1)_=3.84; *P*>0.05; Table 1). This result indicated that the yellow-green phenotype most likely results from a recessive mutation at a single locus and that another locus exists in B73 that can complement this mutation.

**Table 1:** Genetic segregation of F_2_ generations.

Cross	Green leaf	Yellow Leaf	Theoretical Ratio	χ^2^
*elm2*×Mo17	229	66	3: 1	0.9503
*elm2*×Zheng58	220	73	3: 1	0.0011
*elm2*×B73	691	48	15: 1	0.0398

The F_2_ segregation ratio was tested by Chi-square: χ^2^<χ^2^_(0.05, 1)_=3.84, *P*>0.05.

### Determination of pigments and chloroplast development in the *elm2* mutant

 The *elm2* mutation resulted in a reduction in total Chl as well as in Car content (Table 2). The total Chl content in *elm2* was only 16.4% (0.35/2.14) of that found in the wild-type line Zheng58 at the seedling stage; the Chl content in *elm2* increased to 47.2% (1.49/3.16) of the wild type at the tasseling stage (Table 2). The Car content was relatively stable at the two different stages; Car content in *elm2* was maintained at approximately 60% (0.21/0.34; 0.24/0.40) of the level observed in the wild-type line Zheng58 (Table 2). In addition, the *elm2* mutant showed a substantial increase in the Chl *a*/*b* ratio. This ratio was 8.99 at the seedling stage and declined to 4.44 at the tasseling stage (Table 2). These results suggest that the *elm2* mutation mainly affected Chl content rather than Car accumulation, and this delayed de-etiolation during photomorphgenesis.

**Table 2:** Pigment measurements.

	Total Chl (mg g^-1^ FW)	Chl a:b Ratio	Car (mg g^-1^ FW)	Growth Stage
*elm2*	0.35±0.02	8.99±0.26*	0.21±0.01	Seedling
Zheng58	2.14±0.23	3.70±0.06	0.34±0.02	Seedling
Mo17	2.10±0.19	3.69±0.10	0.34±0.03	Seedling
*elm2*	1.49±0.09	4.44±0.17*	0.24±0.01	Tasseling
Zheng58	3.16±0.04	3.43±0.14	0.40±0.03	Tasseling
Mo17	3.19±0.22	3.21±0.11	0.39±0.01	Tasseling

Chl and Car were measured in 80% acetone extracts from the top fully expanded leaves (except the flag leaf) from 4-week-old seedlings or tasseling adults grown in field. Mean and SE values were calculated from five independent determinations. Asterisk indicates significant difference as compared with Zheng58 wild-type at *P*<0.01 (Student’s *t* test).

**Figure 4:**
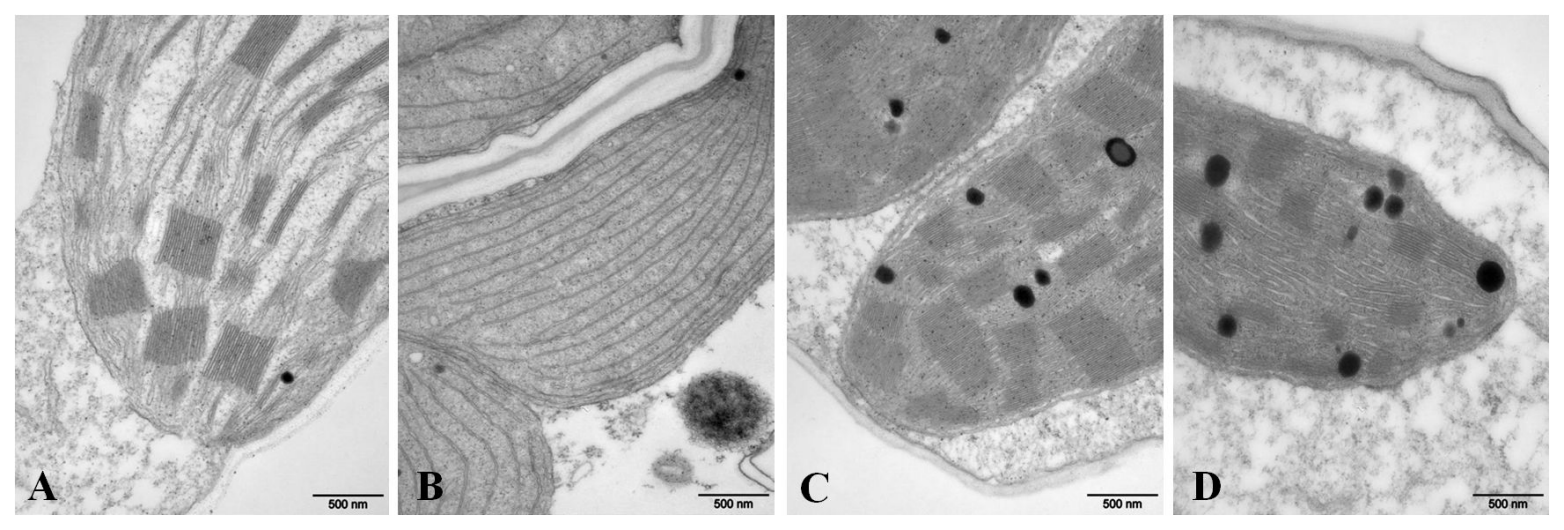
Electron microscope images of chloroplast structures. A and C, Zheng58 wild-type plants at the seedling and tasseling stages, respectively; B and D, *elm2* mutant plants at the seedling and tasseling stages, respectively. Scale bar=500 nm.

Detection of the chloroplast ultrastructure revealed that thylakoid stacking was abnormal in *elm2* compared to the wild-type line Zheng58. The *elm2* mutant specifically stacked non-appressed thylakoids at the seedling stage (Figure 4B). The wild-type line Zheng58 had more and larger granal stacks at the seedling stage, which became much denser at the tasseling stage (Figure 4A, C). Only rare granal stacks appeared in the *elm2* mutant at the tasseling stage (Figure 4D). This indicated that the *elm2* mutation apparently delayed thylakoid stacking, especially grana stacking, during photomorphogenesis.

**Figure 5:**
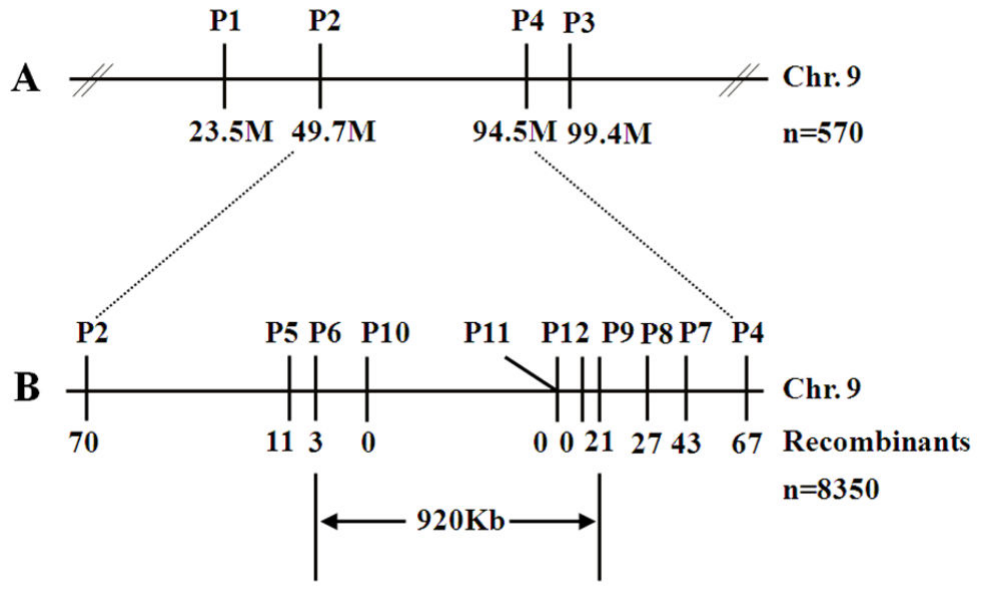
Map-based cloning of *elm2*. The F_2_ population was generated by crossing *elm2* to the wild-type line Zheng58. 8,350 recessive individuals with a yellow-green phenotype were selected for mapping. The candidate region was finally located to a 920 kb region on chromosome 9, which was near the centromere.

To map the *elm2* locus, an F_2_ mapping population was generated from a cross between *elm2* and the wild-type line Zheng58. The *elm2* locus was first mapped to the 9.03 Bin of chromosome 9 between the SSR markers P2 and P4 in 570 F_2_ recessive individuals with yellow-green leaves (Table S1; Figure 5A). To narrow down the mapping region of the *elm2* gene, a larger F_2_ mapping population consisting of 8,350 recessive individuals segregated from more than 33,000 plants were used for fine mapping. Seventy and sixty-seven recombinant individuals were identified from the 8,350 recessive individuals by the use of the markers P2 and P4, respectively. Through further genotyping of the recombinant plants using the markers P5-P12 (Table S1), we localized the *elm2* locus to the interval between markers P6 and P9 and co-segregating with P10-P12 (Table S1; Figure 5B). The candidate region of 920 kb on chromosome 9 was near the centromere; therefore, further mapping would be difficult.

**Figure 6:**
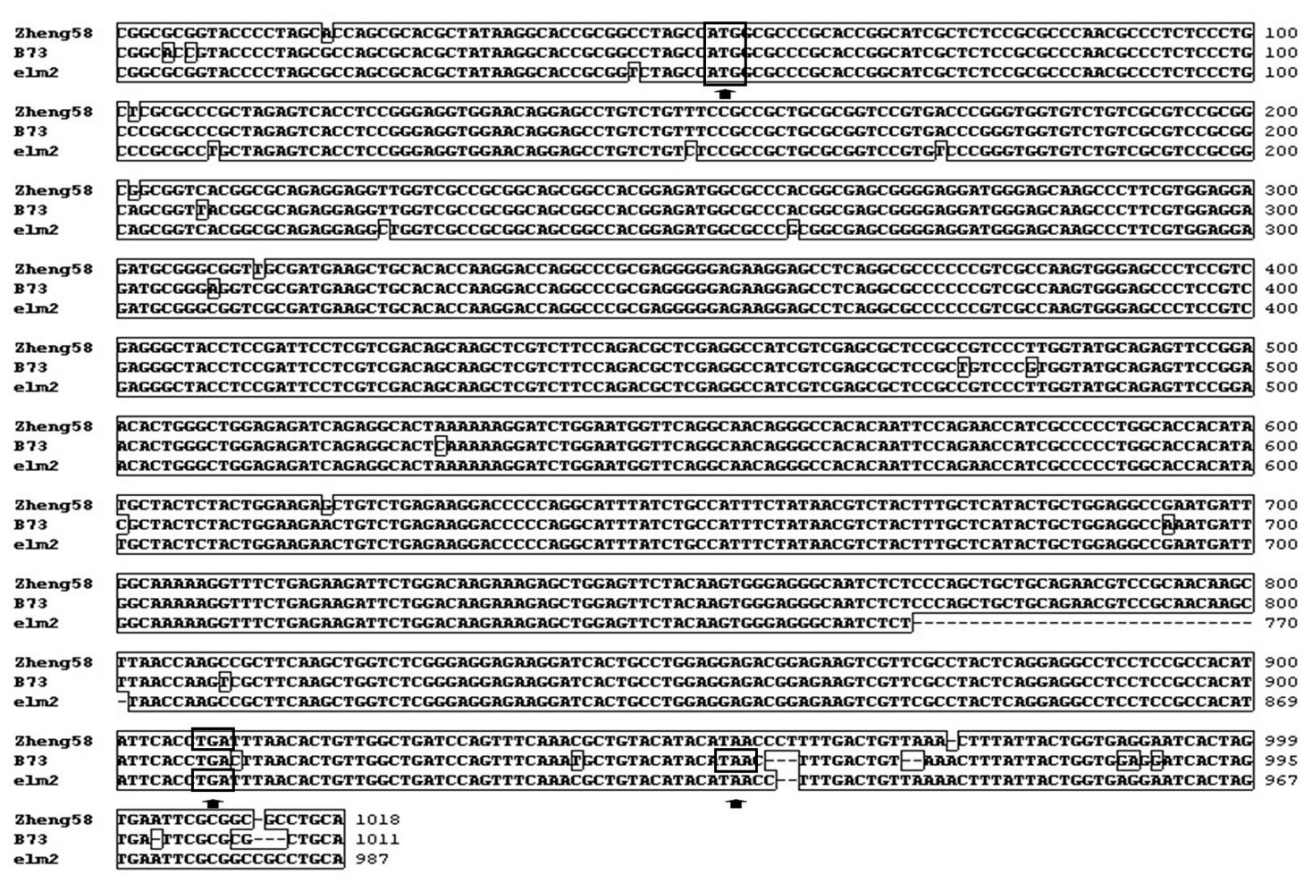
Sequence alignment of *HO1* homologs from *elm2*, B73 and Zheng58. The start (ATG) or stop (TGA/TAA) codons are enclosed with boxes. The dotted lines indicate the 31 bp deletion present in *elm2*. Clustal X software was used for the sequence alignment.

According to the Maizesequence Database (www.maizesequence.org), there are 14 predicted genes within this 920 kb region (Table S2). One of the 14 genes encoded a protein with high homology to *HO1* (Table S2, No 6; Figure S1) [57], which has been extensively analyzed and shown to regulate Chl biosynthesis in Arabidopsis [26], pea [24], tomato [23], and rice [27]. Another *HO1*-like gene was found to reside next to this *HO1* homolog (Table S2, No 7). The remaining 12 candidate genes were not reported to participate in Chl metabolism, or their functions were unknown (Table S2). RT-PCR analysis revealed that no bright bands of the *HO1*-like sequence were detected after 35 cycles amplification in *elm2* and the wild-type line Zheng58 (Figure S2). Genomic sequencing of this *HO1*-like gene showed many inserts and deletions compared to its counterpart in B73 (Figure S3), indicating that this gene most likely does not function during de-etiolation. We then focused on sequencing the *HO1* homolog (Table S2, No 6) in *elm2*; we obtained the complete transcriptional sequence of *HO1* by RT-PCR. The predicted protein consisted of 290 amino acids, while the counterpart in B73 and Zheng58 contained 285 amino acids. An homologous alignment of cDNA with Zheng58 and B73 counterparts showed a 31 bp deletion in *elm2* that caused a translational frame-shift (Figure 6, S4). 

A BLAST (http://blast.ncbi.nlm.nih.gov/Blast.cgi) search with the full-length amino acid sequence of *Elm2* (KC404967) found another *HO1* loci in the B73 genome, on chromosome 6. We also sequenced the paralog on chromosome 6 in *elm2* and Zheng58. The CDS in *elm2* (KC404966) was only 711 bp, which was caused by a premature termination mutation. Amino acid sequence alignment showed that the paralog in *elm2* on chromosome 6 (KC404966) terminated prematurely. In addition, there are at least 3 amino acid variations in the paralog of Zheng58 on chromosome 6 (KC731572) that are conserved in cereal crops (Figure S4).

### Disruption of de-etiolation responses in *elm2* seedlings

It has been reported that *HO* is responsible for converting heme to BV, which is the first committed step in the synthesis of PΦB [12,23-29]. The linear tetrapyrrole chromophore PΦB covalently binds a phytochrome apoprotein and generates the photoactive holophytochrome [30]. According to these mapping and cDNA sequencing results, the *elm2* mutant should show disrupted responses during de-etiolation under monochromatic light if the *ho1* mutation caused the *elm2* phenotype. The de-etiolation responses of *elm2* seedlings indicated that it was developmentally insensitive to red and far-red light, showing a pronounced elongation of the mesocotyl (equivalent to the hypocotyl in Arabidopsis; Figure 2C, D, 3). While under white and blue light conditions, *elm2* showed a moderately elongated mesocotyl corresponding to that observed in the wild-type line Zheng58 (Figure 2B, E, 3), indicating that the blue light receptors do function. Therefore, the morphology of *elm2* suggests a disruption in functional phytochromes. Considering the mapping and cDNA sequencing results, we suggest that the *elm2* mutant was defective in HO1 activity.

### Assay of the endogenous HO activity in *elm2*

As the above results pointed to a *ho1* mutation responsible for the *elm2* phenotype, we directly assayed whether the endogenous level of HO activity had changed. The results demonstrate that the activity in *elm2* was lower than that in the wild-type lines Zheng58 or Mo17. However, the enzyme activity in *elm2* was not inactivated completely and still retained 68% (6.35/9.31) of the activity observed in Zheng58 (Table 3). We assayed the enzyme activity in the F_2_ plants and found a linkage between enzyme activity and leaf color; namely, the enzyme activity in the yellow plants of the F_2_ population (*elm2* cross to Zheng58) was low and the activity in the green plants was high (Table 3). Therefore, we suggest that the *ho1* mutation is responsible for the *elm2* phenotype. However, it is worth noting that the activity assay essentially reflected the ability of the HO to oxidize heme to the BV end product. Other HO members in addition to HO1 could participate in this process in *elm2*, although their effects may be subtler [12,28]. We suspected that the *HO1* mutation could affect other physiological reactions or that the regulation of Chl biosynthesis was combinational, just as many reports demonstrate that HO is a multifunctional enzyme [12,23-28,42-51]. 

**Table 3:** HO activity assay.

Plant	Zheng58	Mo17	*elm2*	F_2_/green	F_2_/yellow
Enzyme Activity (U mg protein^-1^)	9.31±0.70	8.57±0.32	6.35±0.59*	10.70±0.52	6.82±0.68*

One unit of activity was calculated as the quantity of enzyme needed to produce 1 nM BV per 30 min 37°C. Mean and SE values were calculated from four independent determinations. Asterisk indicates significant difference as compared with Zheng58 wild-type or F_2_ individuals with green phenotype at *P*<0.01 (Student’s *t* test).

### Transgenic complementation

The *HO1* homolog from the wild-type line Zheng58 or the *ho1* allele from *elm2*, both driven by the CaMV 35S promoter, were transformed into the corresponding Arabidopsis *hy1-100* mutant. The goal was to determine if the *ELM2* or *elm2* genes could complement the defective responses of the mutant during de-etiolation. To quantify the complementation, we measured the hypocotyl (equivalent to the mesocotyl in maize) length in the transgenic lines under continuous light or constant darkness at 22 °C for 7 d. The light intensity was equivalent to that used when the maize mesocotyls were measured. The results revealed that there were no statistically differences between the Arabidopsis wild-type line Columbia and the transgenic line over-expressing the Zheng58 *HO1* homolog; this indicated that *ELM2* could completely rescue the de-etiolation response (Figure S5, S6). Further measurements demonstrated that there were statistically significant differences between the transgenic lines expressing *ELM2* or *elm2* under continuous white, red or far-red light conditions (Figure S5, S6). Taken together, it appears that the Zheng58 *HO1* homolog could rescue the *hy1-100* phenotype completely. The *ho1* homolog from *elm2* can partially complement this phenotype, although the gene was over transcribed by the CaMV 35S promoter. We therefore suggest that the *ho1* homolog in *elm2* was functionally abnormal and that its mutation caused the defective responses during de-etiolation in *elm2*.

## Discussion

The *elm2* mutant was a spontaneous mutant with yellow-green leaves (Figure 1). Genetic characterization of *elm2* indicated that its mutation resulted from a recessive mutation at a single locus compared with wild-type line Zheng58. Another locus exists in B73 that can complement *elm2* mutation (Table 1). Pigment measurements demonstrated that the Chl level decreased significantly in *elm2*, especially at the seedling stage. Car content was also affected, but was maintained a relatively high level at the two different growth stages in comparison with Zheng58 (Table 2). Therefore, the *elm2* mutation mainly interfered with Chl biosynthesis. Through map-based cloning, the location of the *elm2* allele was found to be within a 920 Kb region on chromosome 9 that contains 14 predicted genes (Figure 5). Among these 14 candidate genes, the *HO1* homolog was most likely related to the *elm2* phenotype; this was determined according to previous reports [23-25,27]. cDNA sequencing of this homolog showed a 31 bp deletion that resulted in a frame-shift mutation. The predicted CDS encodes 290 amino acids and is 15 base pairs longer than the CDSs found in Zheng58 and B73 (Figure 6).

It was reported that *HO* belongs to the biosynthesis pathway of the phytochrome chromophore; its mutation would cause a phytochrome deficiency and show a disruption in the de-etiolation response [12,25]. Furthermore, *HO1* is responsible for the majority of BV synthesis during photomorphogensis. The other gene family members play a role in synthesizing BV, but their effects are subtler [12,25,26,28]. When grown under continuous red or far-red light conditions, *elm2* seedlings showed elongated mesocotyls as expected (Figure 2). This result indicated a phytochrome deficiency in *elm2*. Because phytochrome apoproteins are encoded by a multigene family with at least six copies in maize [18-22] that all bind the same chromophore [30,31], the chromophore synthetic pathway is more likely to cause a functional phytochrome deficiency. Considering this with the cDNA sequencing results (Figure 6, S4), we suggest that the *elm2* mutant was defective in HO1 activity.

An assay of the endogenous HO activity revealed that the HO level in *elm2* was significantly decreased compared with the wild-type lines Zheng58 and Mo17 (Table 3). Upon further assaying of the F_2_ plants, we found there was a linkage between enzyme activity and leaf color; the enzyme activity in the yellow plants of F_2_ population was low, while the activity in the green plants was high (Table 3). The linkage indicated that the *elm2* phenotype was most likely caused by a defect in HO activity. Genetic transformation showed that over-expressing the *HO1* homolog from Zheng58 could completely rescue the defective responses during de-etiolation of the corresponding Arabidopsis *hy1-100* mutant (Figure S5, S6). Arabidopsis plants over-expressing the mutant *ho1* homolog from *elm2* showed statistically significant differences compared to plants over-expressing *HO1* homolog from Zheng58 under continuous white, red or far-red light conditions (Figure S5, S6). Taken together, we suggest that *elm2* encoded a mutant *ho1* homolog.

A BLAST search with the full-length amino acid sequence of *Elm2* (KC404967) found two *HO1* loci in B73 genome, with one loci on chromosome 9 (within our located region) and another on chromosome 6. The F_2_ segregation ratio of crosses to B73 and Zheng58 indicated that the two paralogs in B73 and the one paralog in Zheng58 could function normally (Table 1). The two paralogs in *elm2* are both defective, which is consistent with the subsequent cDNA sequencing results. The complete cDNA of *ho1* in *elm2* on chromosome 9 (KC404965) showed a 31 bp deletion that resulted in a translational frame-shift (Figure 6). The *elm2* CDS on chromosome 6 (KC404966) was only 711 bp; this truncated sequence was caused by a premature termination mutation (Figure S4). Although the CDS length of the paralog in Zheng58 on chromosome 6 (KC731572) was as expected, there are at least 3 amino acid variations in this paralog that are conserved in cereal crops (Figure S4). Because maize is often cross-pollinated, long-term self-pollination for breeding may result in genetic changes [58]. Deleterious mutations are accumulated until the persistence of the phenotype is threatened [59]; this phenomenon is usually referred to as inbreeding depression [60]. This phenomenon is commonly observed in the conservation of homozygous inbred lines by self-pollination [61]. The genetic variation in the *HO1* gene in maize presents a good example of in-depth inbreeding depression. In addition, the *HO1* gene belongs to a small family [12]; therefore, the genetic divergency can potentially be used as a selection index in future breeding programs to reduce the time and effort required in large-scale field tests [58].

Examination of the chloroplast ultrastructure revealed that non-appressed thylakoids were specifically stacked in *elm2* at seedling stage (Figure 4B). Even at the tasseling stage, only rare granal stacks appeared in *elm2* (Figure 4D). This phenotype was different than that observed in Chl biosynthetic mutants, such as the Arabidopsis *porB* and *porC* single mutants and the *porB porC* double mutant [7] and the rice *ygl1* mutant (encoding Chl synthase) [53]. Chloroplasts in these mutants contained primarily unstacked thylakoids, with occasional distributed double membrane stacks. While the granal stacks in the rice *824ys* mutant (encoding divinyl reductase) [62] appeared relatively normal, they appeared was less dense than in the wild type. Previous studies have shown that Chl biosynthesis appears to be feedback-inhibited by HEMA activity; this is performed by controlling the heme level, as HEMA is the first enzyme committed to tetrapyrrole biosynthesis [40,41]. The abnormal grana stacking in *elm2* indicated that the regulation of Chl biosynthesis by *HO1* may be a combinational result, which is achieved by coordinating the inhibition of HEMA activity with the regulation of grana stacking. The *ho1* mutants will otherwise show grana stacking characteristics similar to the Chl biosynthetic mutants. The specific regulation of grana stacking most likely was achieved by regulating the expression of *Lhcb* via phytochrome signaling [63]. 

In conclusion, we isolated a *ho1* homolog by map-based cloning from the maize *elm2* mutant and characterized its function comprehensively. The 31 bp deletion in the *ho1* gene resulted in a decrease in endogenous HO activity and disrupted the de-etiolation responses to red and far-red signal; this resulted in a yellowish phenotype. The divergence of the *HO1* gene in different inbred lines is a good example of in-depth inbreeding depression, and this gene has potential as a selection index in future breeding programs. Furthermore, the specific stacking of non-appressed thylakoids demonstrated that *HO1* may also regulate grana stacking, which contributed to the yellowish phenotype observed in the *ho1* mutants.

## Supporting Information

Figure S1 **Phylogenetic analysis of ELM2 and the previously reported HO enzymes.** The neighbor-joining method designed in the MEGA 4.0 software program was used to construct the phylogenetic tree. The branch length indicates the extent of divergence according to the scale at the bottom.
(TIF)

Figure S2 **RT-PCR analysis of the 14 candidate genes.** Z58-D and *elm2*-D are seedlings (above the mesocotyl) from the Zheng58 wild-type and the *elm2* mutant, respectively, under constant darkness for 7 d; Z58-W and *elm2*-W are seedlings (above the mesocotyl) from the Zheng58 wild type and the *elm2* mutant, respectively, under continuous white light for 7 d. The 14 candidate genes are labeled G1-G14. *β-actin* was amplified as a control.
(TIF)

Figure S3 **Genomic sequence comparisons of the *HO1*-like gene on chromosome 9 in *elm2* and B73.** The predicted exons are underlined. The start codon and termination codon are indicated by black arrows. The predicted CDS in *elm2* terminated prematurely; the length in *elm2* is 552 bp whereas its counterpart in B73 is 729 bp.
(TIF)

Figure S4 **Amino acid sequence comparison of HO1 paralogs in maize, sorgum, and rice.** The black triangles denote the amino acid mutations in Zheng58-Chr6. The white triangle marks the premature termination in elm2-Chr6. The black arrow indicates the frame shift in elm2-Chr9.
(TIF)

Figure S5 **De-etiolation responses in transgenic lines.** The seedlings are, from left to right, the Columbia wild-type, the *hy1-100* mutant, the transgenic lines *hy1-100*/*ZmHO1*-ox (with *HO1* from Zheng58), and *hy1-100*/*Zmho1*-ox (with *ho1* from *elm2*). A, constant darkness; B, white light; C, red light; D, far-red light; E, blue light. The sample size is 15-18 seedlings per treatment/genotype. Scale bar=1 mm.
(TIF)

Figure S6 **Statistical measurement of hypocotyl length in transgenic lines.** *hy1-100*/*ZmHO1*-ox and *hy1-100*/*Zmho1*-ox are transgenic lines with the *HO1* sequence from Zheng58 and *ho1* from *elm2*, respectively. The sample size is 15-18 seedlings per treatment/genotype. Bars denoted by the different letters were different significantly at *P*<0.01 according to Tukey's multiple range test.
(TIF)

Table S1 **The PCR-based molecular markers designed for fine mapping.**
(PDF)

Table S2 **Gene annotation within the identified region.**
(PDF)
