# Supplementary figures and images for: Chlorophyll Deficiency in the Maize elongated mesocotyl2 Mutant Is Caused by a Defective Heme Oxygenase and Delaying Grana Stacking
Source: PLoS One. 2013 Nov 11;8(11):e80107. doi: 10.1371/journal.pone.0080107 (PMC3823864; doi:10.1371/journal.pone.0080107)

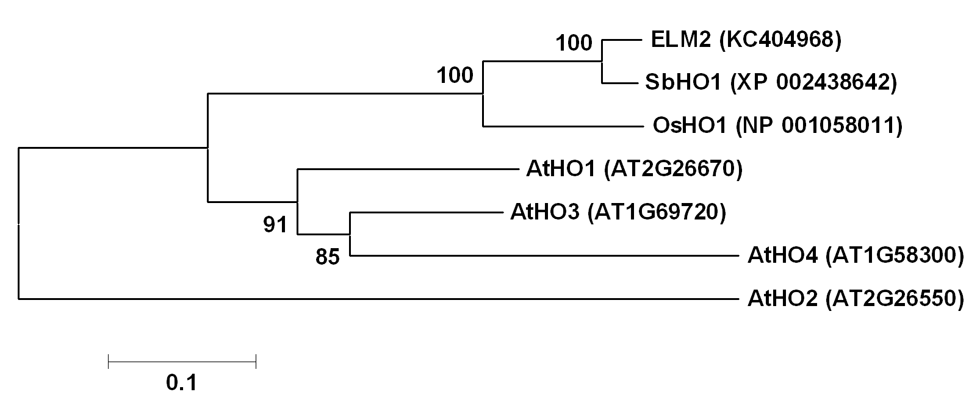

Supplement: Figure S1 — Phylogenetic analysis of ELM2 and the previously reported HO enzymes. The neighbor-joining method designed in the MEGA 4.0 software program was used to construct the phylogenetic tree. The branch length indicates the extent of divergence according to the scale at the bottom. (TIF) [file pone.0080107.s001.tif]

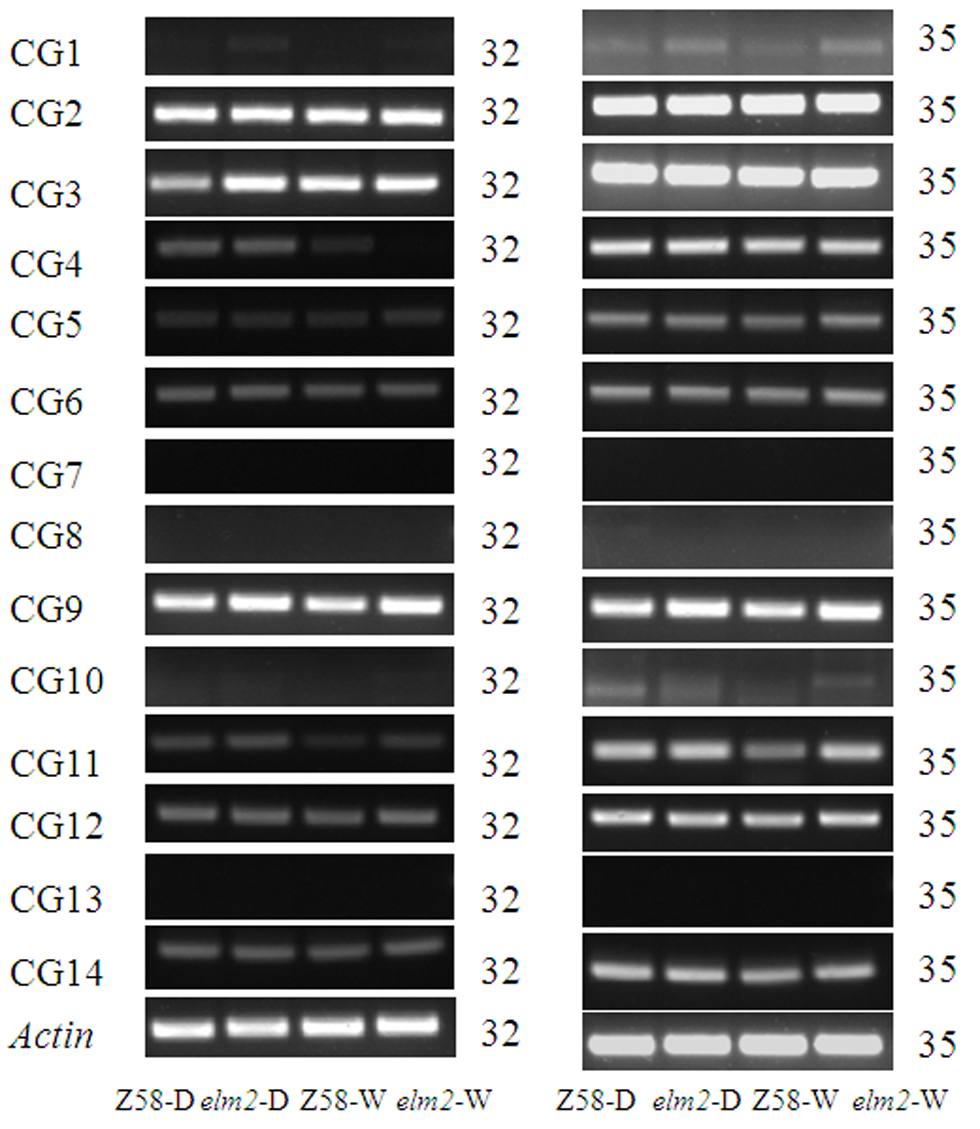

Supplement: Figure S2 — RT-PCR analysis of the 14 candidate genes. Z58-D and elm2-D are seedlings (above the mesocotyl) from the Zheng58 wild-type and the elm2 mutant, respectively, under constant darkness for 7 d; Z58-W and elm2-W are seedlings (above the mesocotyl) from the Zheng58 wild type and the elm2 mutant, respectively, under continuous white light for 7 d. The 14 candidate genes are labeled G1-G14. β-actin was amplified as a control. (TIF) [file pone.0080107.s002.tif]

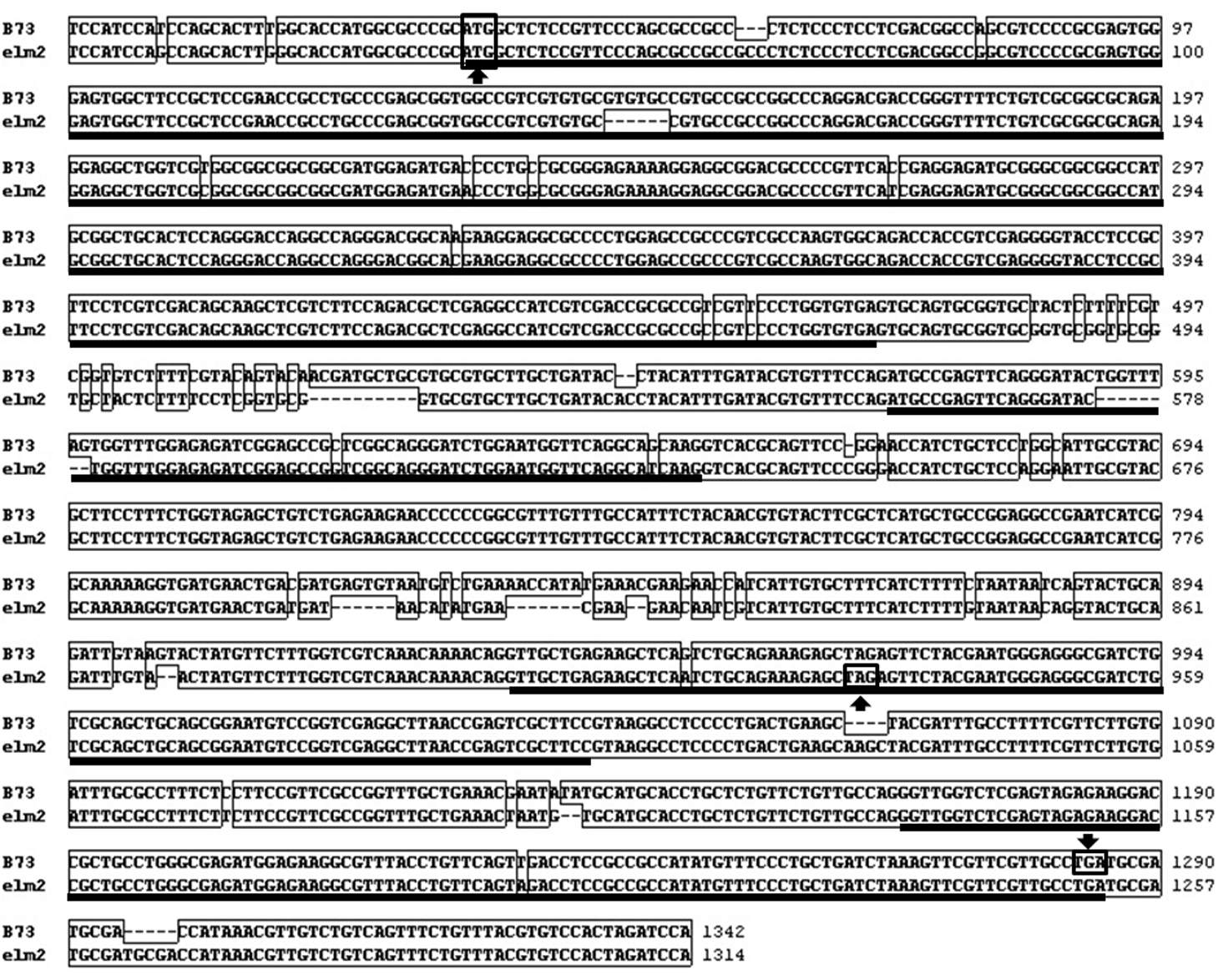

Supplement: Figure S3 — Genomic sequence comparisons of the HO1-like gene on chromosome 9 in elm2 and B73. The predicted exons are underlined. The start codon and termination codon are indicated by black arrows. The predicted CDS in elm2 terminated prematurely; the length in elm2 is 552 bp whereas its counterpart in B73 is 729 bp. (TIF) [file pone.0080107.s003.tif]

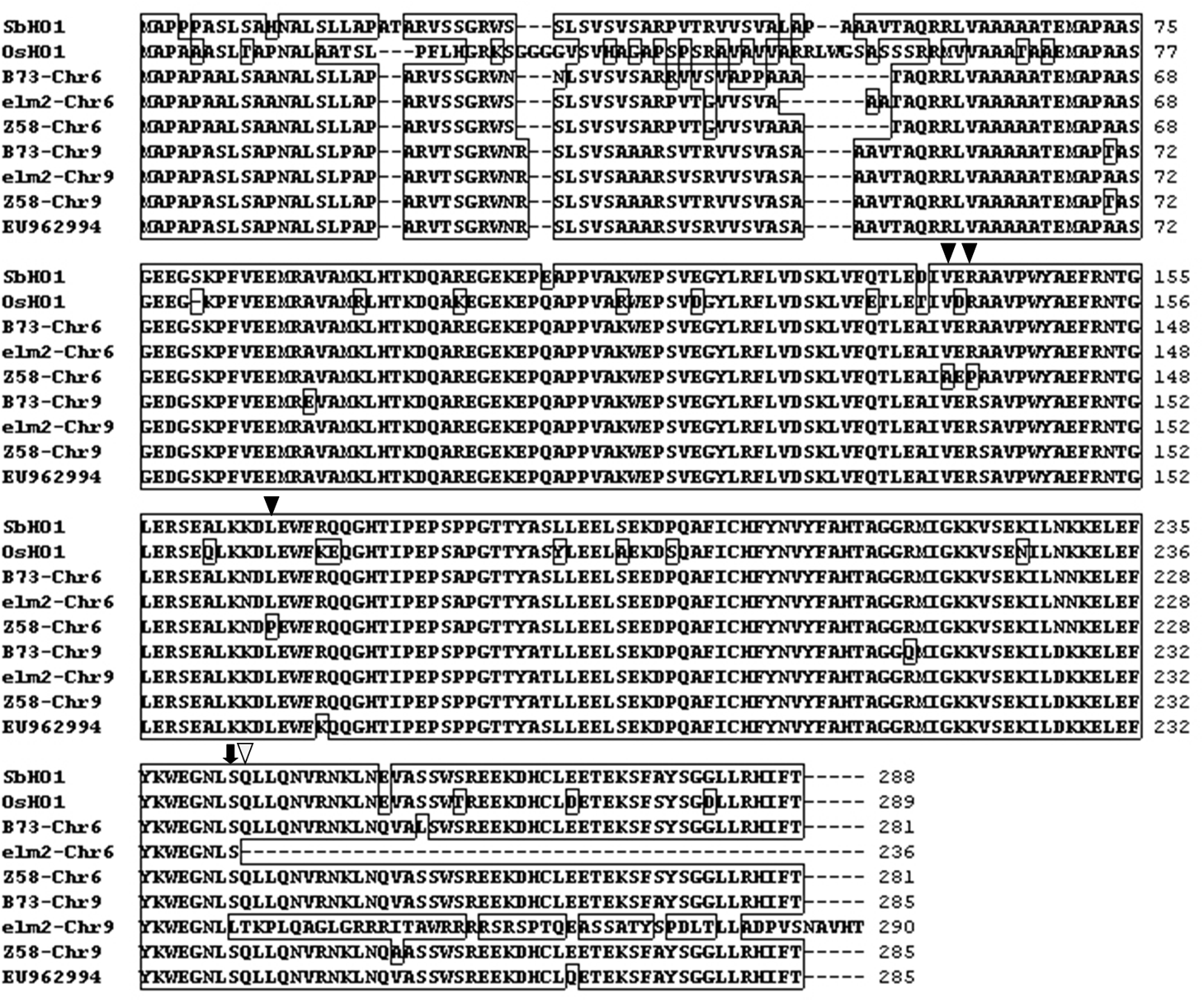

Supplement: Figure S4 — Amino acid sequence comparison of HO1 paralogs in maize, sorgum, and rice. The black triangles denote the amino acid mutations in Zheng58-Chr6. The white triangle marks the premature termination in elm2-Chr6. The black arrow indicates the frame shift in elm2-Chr9. (TIF) [file pone.0080107.s004.tif]

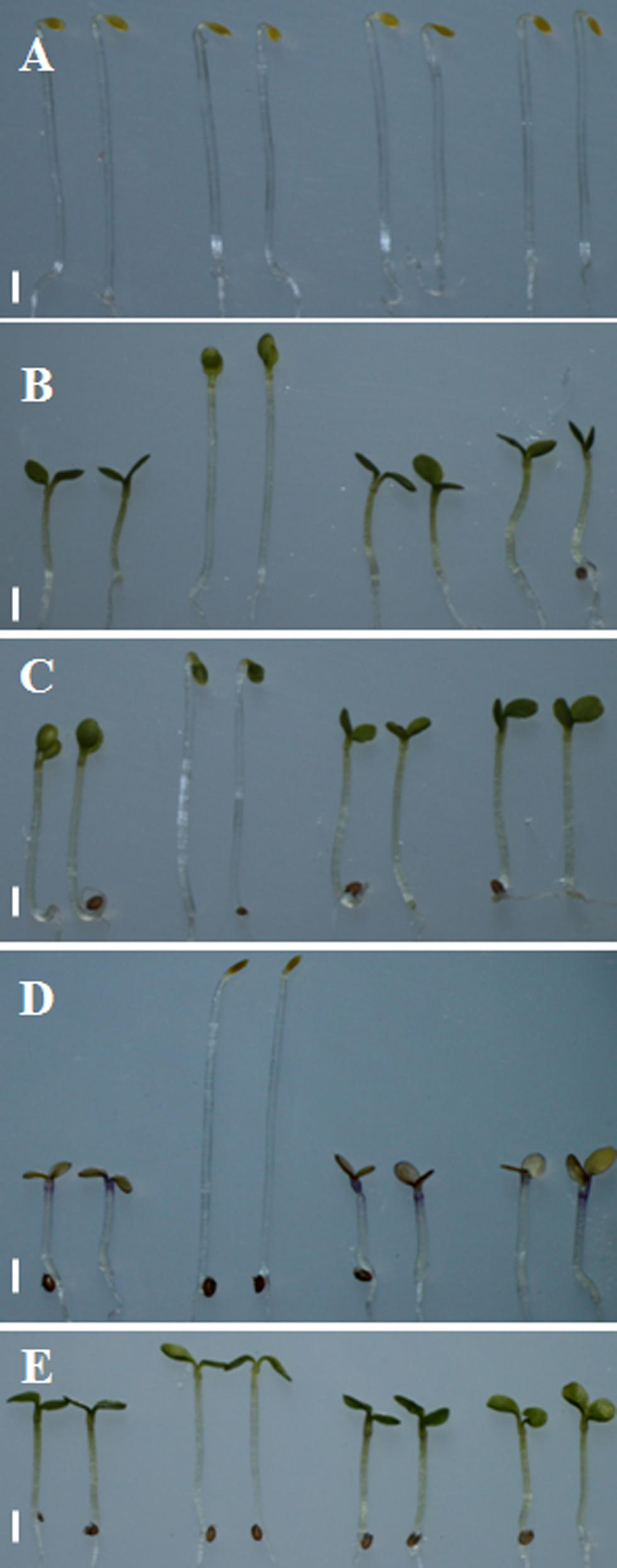

Supplement: Figure S5 — De-etiolation responses in transgenic lines. The seedlings are, from left to right, the Columbia wild-type, the hy1-100 mutant, the transgenic lines hy1-100/ZmHO1-ox (with HO1 from Zheng58), and hy1-100/Zmho1-ox (with ho1 from elm2). A, constant darkness; B, white light; C, red light; D, far-red light; E, blue light. The sample size is 15-18 seedlings per treatment/genotype. Scale bar=1 mm. (TIF) [file pone.0080107.s005.tif]

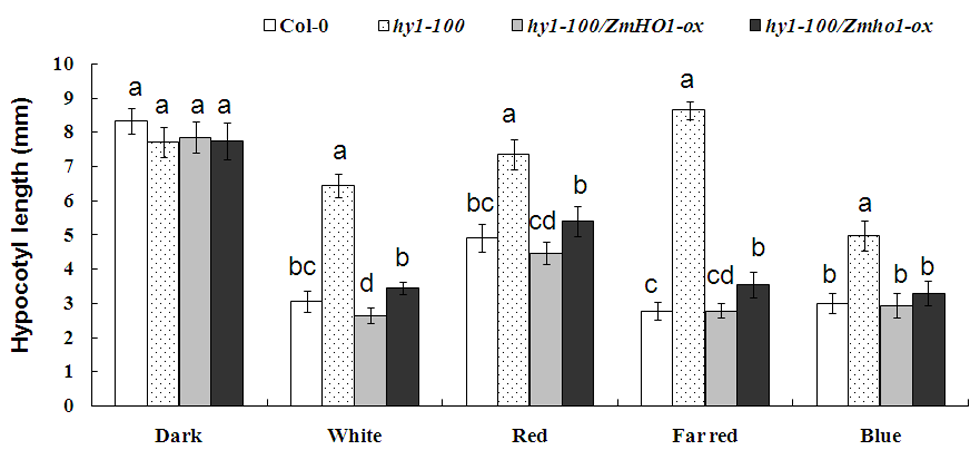

Supplement: Figure S6 — Statistical measurement of hypocotyl length in transgenic lines. hy1-100/ZmHO1-ox and hy1-100/Zmho1-ox are transgenic lines with the HO1 sequence from Zheng58 and ho1 from elm2, respectively. The sample size is 15-18 seedlings per treatment/genotype. Bars denoted by the different letters were different significantly at P<0.01 according to Tukey's multiple range test. (TIF) [file pone.0080107.s006.tif]
