# Supplementary material for: Chlorophyll Deficiency in the Maize elongated mesocotyl2 Mutant Is Caused by a Defective Heme Oxygenase and Delaying Grana Stacking
Source: PLoS One. 2013 Nov 11;8(11):e80107. doi: 10.1371/journal.pone.0080107 (PMC3823864; doi:10.1371/journal.pone.0080107)
Supplement: Table S1 — The PCR-based molecular markers designed for fine mapping. (PDF) [file pone.0080107.s007.pdf]

Table S1. The PCR-based molecular markers designed for fine mapping

No	Primer Name	Type	Restriction Enzyme	Primer Pairs	Originated BAC
P1	Phi027	SSR		CACAGCACGTTGCGGATTTCTCT<br>GCGTACGTACGACGAAGACAC	AC190908
P2	Bnlg127	SSR		CATGTATACGAGAAGCACCCTAT<br>ATCGTAACTCAGCGGTTTGTG	AC210057
P3	Umc1743	SSR		TGGACTTCGAAAATTCTCTTCAGC<br>GAGAGGAGGAGCTTCACGAGC	AC215636
P4	AC196277-5	SSR		TCAACTTAAGGTCATGCCCTAG<br>CGAGCTGAGCATGGCTAGAG	AC196277
P5	Umc1191	SSR		AAGTCATTGCCCAAAGTGTTGC<br>ACTCATCACCCCTCCAGAGTGTC	AC200537
P6	AC200537-C	CAPS	Hind III	TGCTCGATTCCAAGTATGA<br>AGCAGCAGCATTACCAAA	AC200537
P7	AC208971-5	SSR		TTCCAAACAGAACCGACC<br>CGAAAGGATAAGACTAGAGC	AC208971
P8	AC202130-6	SSR		GAGCATTCATTAGCACCG<br>CTCAGCGCCATATCACAT	AC202130
P9	AC202130-16	SSR		TCCACCACGTAATAGGAAATA<br>ACCTGTGCAAGGGCAACT	AC202130
P10	AC188985-5	Indel		TATGGCCGAGACTGTGGC<br>GACGATGGCGGAATGTTG	AC188985
P11	AC215185-9	Indel		CAGTCCCATCTCCAGCAA<br>TCCGGGCAGAGCAACTAG	AC215185
P12	AC215185-12	Indel		ATCCCTGGCATCCCGTCG<br>GATCTGGTGGGGCGTTCG	AC215185
