# Supplementary material for: Chlorophyll Deficiency in the Maize elongated mesocotyl2 Mutant Is Caused by a Defective Heme Oxygenase and Delaying Grana Stacking
Source: PLoS One. 2013 Nov 11;8(11):e80107. doi: 10.1371/journal.pone.0080107 (PMC3823864; doi:10.1371/journal.pone.0080107)
Supplement: Table S2 — Gene annotation within the identified region. (PDF) [file pone.0080107.s008.pdf]

Table S2. Gene annotation within the located region

No	Transcript ID	Location on Chromosome 9	Protein Length (aa)	Predicted Function	Primer Pairs for RT-PCR
1	GRMZM2G310362	88,725,670-88,729,468	643	Polyadenylate binding protein	GAATGAATGGTAGGATTGTTGGG；<br>GCATCATAACGGGAGAGATAGGC
2	GRMZM2G009995	88,729,883-88,735,673	765/483	Serine/threonine-protein kinase	CGTTTCTGAATGCGTGACAAATGGA；<br>TCTTCATGGACAGCAAATGGGGT
3	GRMZM2G019586	88,793,064-88,796,135	346/382	Tyrosine-protein kinase	TGTCTGGGGTTCGGTCAAAG；<br>GGGTCCTCCAATTCTGTCGC
4	GRMZM2G019872	88,798,886-88,800,768	343/356	Unknown	GCTCAAAGGCAAGCTGGGATT；<br>TTGGACAAGAAGTCGTAGGCG
5	GRMZM2G099642	88,874,336-88,876,199	344	Alcohol dehydrogenase	GGGGAGAAGGTGTTCGTGTCG；<br>CGGCCATAGGTATTCATGTTGG
6	GRMZM2G101004	88,918,347-88,927,536	285/29	Heme oxygenase	GCGGTTGCGATGAAGCTG；<br>GTGGCCCTGTTGCCTGAA
7	GRMZM2G157936	88,985,396-88,986,632	242	Heme oxygenase-like	GTCGTTCCCTGGTATGCCGAGTTCA；<br>CGGAGGTCAACTGAACAGGTAAACG
8	GRMZM2G069169	89,016,989-89,017,775	209	Unknown	CCCCAAGGTCATCCACGTC；<br>GGAACAACATCAGCCCCGAC
9	GRMZM2G058336	89,198,117-89,199,404	260	RNA polymerase	CACTGACGCCGTAACCACT；<br>CCCTCCATCATCCCTCCCT
10	GRMZM5G853065	89,326,235-89,327,722	182/255/175	Glucose/ribitol dehydrogenase	CTGTAGGAGTAAGAGTAGGGCTGGA；<br>TCGGCAGCGAAGCTCACC
11	GRMZM5G827342	89,329,738-89,330,633	55	Unknown	GTTGCACGAGCACGAGGGACAC；<br>GTCTTCAGCATTCCTTGTTACCATA

12	GRMZM2G035579	89,332,725-89,333,577	117	Unknown	GATGGAAAAGGAGACTCTAATGAT；CGCCTGCTCTATCACTGTTACT
13	GRMZM2G083655	89,392,972-89,394,125	155/156	Unknown	GCGGACAGATCGTGGAGAC；
					GCTCCTTCTGACGGATGATGTA
14	GRMZM2G570791	89,546,232-89,546,523	49	Unknown	CAAGAGAAATAAACGATTCAATGAT；
					GCGTGTGATACATGTTCCTTCTA
